# Composite lymphoma in the anterior mediastinum: a case report and review of the literature

**DOI:** 10.1186/1746-1596-6-60

**Published:** 2011-07-06

**Authors:** Guohua Yu, Lingling Kong, Guimei Qu, Qian Zhang, Wei Wang, Lei Jiang

**Affiliations:** 1 Department of Pathology, Affiliated Yantai Yuhuangding Hospital, Medical College of Qingdao University, No.20, Yuhuangding East Road, Yantai, 264000, China; 2 Department of Pathology, Binzhou Medical University, No.346, Guanhai Road, Yantai, 264003, China

**Keywords:** Composite lymphoma, nodular sclerosing Hodgkin lymphoma, diffuse large B-cell lymphoma, immunohistochemistry, in situ hybridization

## Abstract

We recently encountered an unusual case of Composite lymphoma (CL) in the anterior mediastinum arising in a 37-year-old woman who presented initially with continuous pain in the right shoulder and chest. The woman had been suffered from continuous pain for three months before she went to our department of cardiovascular surgery. Chest computed tomography scan revealed the oval space-occupying lesion of anterior mediastinum. Surgery was performed and the disease was diagnosed pathologically as CL which composed of nodular sclerosing Hodgkin lymphoma and diffuse large B-cell lymphoma, via hematoxylin-eosin (H&E), immunohistochemical staining and in situ hybridization.

## Background

Composite lymphoma (CL), which is defined as the coexistence of two morphologically and phenotypically distinct types of lymphoid neoplasms occurring in a single anatomic organ or tissue, is unusual [1,2]. The combination might include Hodgkin lymphoma (HL) with B-cell or a T-cell non-Hodgkin lymphoma (NHL), B-cell NHL with T -cell NHL, or two distinct B-cell or T-cell NHLs at the same anatomic site [2-6]. The concept of "composite lymphoma" was first put forward by Custer RP to expatiate the occurrence of more than one histological type of lymphoma in the same patient [7]. The incidence of CL is low, varying from 1% to 4.7% [8]. In our present study, we report a case of composite nodular sclerosing Hodgkin lymphoma (NSHL) and diffuse large B-cell lymphoma (DLBCL) in the anterior mediastinum. The clinical, histopathologic, immunohistochemical features and the clinical prognosis are discussed.

## Case presentation

The 37-year-old woman presented with a three-month history of continuous pain in the right shoulder and chest. She was taken to our department of cardiothoracic surgery. Physical examination was remarkable for palpable lymph nodes in the bilateral supraclavicular fossae. Chest computed tomography (CT) revealed an oval mass located in the anterior mediastinum. Circumambient lung tissue was involved. Haematological findings were: WBC 15.40 × 10^9^/L, platelets 364 × 10^9^/L, prothrombin time 10.3 sec. Her erythrocyte sedimentation rate (ESR), blood biochemistry, electrolytes, blood urea nitrogen and serum creatinine, urine analysis and the endocrine profile were within normal range except hemoglobin (106 g/L) and tumor specific growth factor (67 U/ML). The abdominal ultrasonography did not reveal any other abnormality.

**Figure 1:**
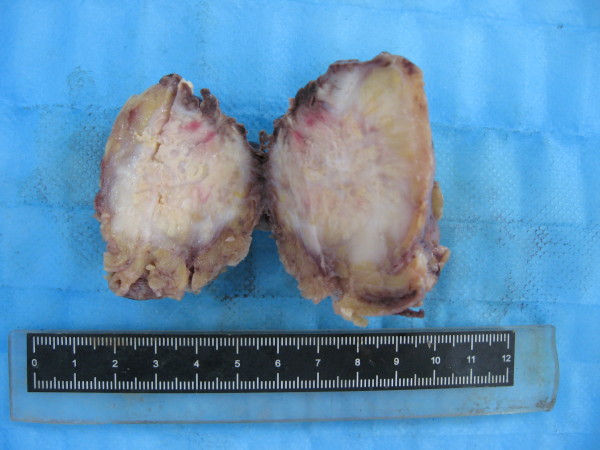
**Gross morphology of the tumor**. The cut section of the tumor is off-white with obscure boundaries and hard in consistence. Volume of the tumor is 7 cm × 4.5 cm × 2.5 cm.

The surgery was performed with the patient under general anesthesia and left lateral decubitus position. The procedure revealed an oval tumor, volume of which was 7 cm × 4.5 cm × 2.5 cm, in the anterior mediastinum and circumambient lung tissue was involved. Pathologic findings during surgery showed that the tumor was off-white with obscure boundaries and hard in consistence (Figure 1). The result of pathology hinted that the tumor maybe malignant lymphoma. The tumor and circumambient lung tissue which was involved were excised.

**Figure 2:**
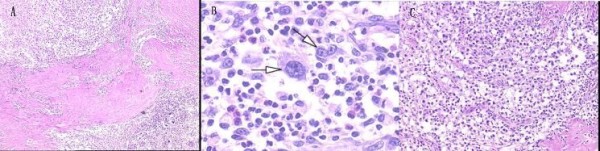
**Histological examination**. (A)The tumor is nodular and different nodules are separated by collagen fibers. Different nodules show different histologic features (H&E × 40). (B) Pleomorphic cellular infiltrate containing lymphocytes, eosinophils, neutrophils and large Reed-Sternberg cells (arrows) (H&E × 400). (C) Other nodules display sheets of relatively uniform large lymphoid cells with typical morphologic features of large cell lymphoma (H&E × 100).

**Figure 3:**
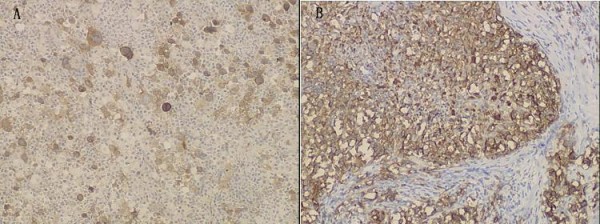
**Immunohistochemical features of the tumor**. (A)Immunohistochemical study demonstrates that Reed-Sternberg cells are positive for CD30 (Envision × 100). (B) Immunohistochemical study shows that diffuse large B-cell lymphoma cells is positive for CD20 (Envision × 100).

Histology showed the tumor was nodular and different nodules were separated by collagen fibers (Figure 2A). There were two morphologically and immunophenotypically distinct components. The great mass of nodules showed a pleomorphic cellular infiltrate containing lymphocyte, eosinophile granulocyte, neutrophile granulocyte and numerous large Hodgkin/Reed-Sternberg cells. The Reed-Sternberg cells were positive for CD30, CD15 and MUM1 but negative for CD20, CD79a, CD3, Pax-5, CD68, bcl-6, CD10, CD45, Igκ, Igλ and epithelial membrane antigen (Figure 2B, Figure 3A). Other nodules displayed sheets of relatively uniform large lymphoid cells with typical morphologic features of large cell lymphoma which showed uniform expression of CD20, CD79a, MUM1, CD45, Igλ, Pax-5 and absence of CD30, CD15, CD3, CD10, bcl-6, Igκ, CD68 and epithelial membrane antigen (Figure 2C, Figure 3B). All the primary antibodies are listed in Table 1. Neither cell population showed makers of EBV infection by EBER in situ hybridization (PanPath Company, Amsterdam, Netherlands). On the basis of histomorphology at light microscopy, the presence of immunohistochemical staining and in situ hybridization, a diagnosis of CL, combination of NSHL and DLBCL, in the anterior mediastinum was made. The patient received six courses of CHOP chemotherapy (a course of treatment every two weeks) and twenty three times radiotherapy (Gross Tumor Volume, GTV = 40Gy/20f; Planning Target Volume, PTV = 36 Gy/20f). After treatment, lymph nodes in the bilateral supraclavicular fossa disappeared. The total follow-up period was thirty three months after surgery. The repeated CT scans, abdominal ultrasonography and tumor specific growth factor every three months revealed no recurring or residual lesion.

**Table 1:** Antibodies employed in the immunohistochemistry applied

Antibody	Clone	Source	Dilution
Anti CD3	SP7	Lab Vision	1:100
Anti CD20	L26	Lab Vision	1:100
Anti CD79a	SP18	Lab Vision	1:100
Anti CD45	PD7/26+2B11	Lab Vision	1:100
Anti CD10	56C6	Lab Vision	1:100
Anti CD15	Carb-3	Lab Vision	1:100
Anti CD30	Ber-H2	Lab Vision	1:100
Anti CD68	KP1	Lab Vision	1:100
Anti MUM1	MUM1p	Lab Vision	1:100
Anti Pax-5	SP34	Lab Vision	1:100
Anti Bcl-6	LN22	Dako	1:100
Anti Igk	L1C1	Lab Vision	1:100
Anti Igλ	LAM03+HP6054	Lab Vision	1:100

## Discussion

Clinically, manifestation of CL is similar with that of ordinary lymphoma. The true incidence of CL occurring in the same tissue is difficult to estimate because of the pathologist and the classification system used. In the study of more than one thousand of cases for Working Formulation of NHL, the incidence of CL varied between 1 and 4.7% [6,9]. The combination of classical Hodgkin lymphoma and non-Hodgkin lymphoma coexisting in the same tissue is rare and much more uncommon than other combinations. To our best knowledge, Kim et al firstly reported the patient with CL consisting of an admixture of HL and NHL in the left axillary node [10]. According to our literature search, only six cases showing combination of classical Hodgkin lymphoma and DLBCL within the same site simultaneously were described [11-15]. The clinical data of all previously published cases of classical Hodgkin lymphoma associated with DLBCL was listed in Table 2.

**Table 2:** Clinical data of previously published cases of classical Hodgkin lymphoma with DLBCL

No./Series	Age (years)	Sex	Site of biopsy	Clinical Presentation	Subtype of cHL	Treatment	Follow-up Months	Status
**1**/Paulli et al 1992	37	Male	LN in the right SF	Intermittent fever and malaise	NS	Eight cycles of pro-MACE-CytaBOM	23	ANED
**2**/Huang et al 2006	56	Male	Small intestine	Bowel perforation	NS	The lesion was resected	6 days after surgey	DOD
**3**/Bellan et al 2002	29	Female	Laterocervical LN	Left LL extending to the SF	NS	MACOP-B chemotherapy for 8 weeks and transplant of autologous stem cells	30	ANED
**4**/Rosenquist et al 2004	74	Female	LN in the right inguinal	Abdominal pain	MC	Six cycles of CHOP	12	ANED
**5**/Hell et al 1995	NA	NA	LN	NA	NS	NA	NA	NA
**6**/Hell et al 1995	NA	NA	LN	NA	NS	NA	NA	NA
**7**/Present case	37	Female	mediastinum	continuous pain in the right shoulder and chest	NS	six courses of CHOP chemotherapy and twenty three times radiotherapy	33	ANED

Abbreviations: cHL, classical Hodgkin Lymphoma; NS, nodular sclerosis; MC, mixed cellularity; LN, lymph node; SF, supraclavicular fossa; LL, laterocervical lympadenopathy; ANED, alive with no evidence of disease; DOD, died of disease; NA, not available.

Lymphoma, generally, is defined as monoclonal proliferation of lymphocyte (T cell, B cell or natural killer cell) while polyclonal hyperplasia is generally regarded as reactive lymphoid proliferation. However, the pathogenesis of CL is not clear. Many studies of CL using molecular techniques have proved true biclonality as well as a common origin from the same clonal progenitor cell and suggested different biological mechanisms in different tumors [4,13,14,16-18]. As far as the combination of HL and B-cell NHL is concerned, the most common form is nodular lymphocyte predominance Hodgkin lymphoma (NLPHL) with large cell lymphoma [6]. The both components may occur synchronously and successively. More and more researchers swing the pendulum towards that HL and B-cell NHL coexisting in the same tissue might originate from a common precursor by molecular techniques [13,14,19]. Bräuninger et al studied CL from one patient (classic HL and a follicular lymphoma in the same lymph node). A V gene rearrangement and some somatic mutations were shared by Reed-Sternberg cells and follicular lymphoma cells, which provided proof of a common B-cell precursor [16]. Schmitz et al summarized six cases of such CL in which the HLs were combined in two cases with follicular lymphoma, and in one case each with B-cell chronic lymphocytic leukemia, marginal zone lymphoma, mantle cell lymphoma and DLBCL. The composite lymphomas had been analyzed by single-cell PCR analysis for rearranged IgV genes and a clonal relationship was confirmed [16,20,21]. In the cases of NLPHD with associated LBCL, the presence of a clonal population in the NLPHD component that was identical or related to the neoplastic clone of the associated LBCL has been found. In addition, in situ hybridization has shown that both L&H cells and LBCL cells expressed the same type of the immunoglobulin light chain messenger RNA. Single L&H cells, the Reed-Sternberg cells variants in NLPHD, were isolated from immunostained tissue sections by micromanipulation, and the IgH complementarity determining region (CDR) III of the cells was amplified by PCR. The sequences from the L&H cells also were related to those from the corresponding LBCL cells. All of these findings strongly suggested that NLPHD was related to the corresponding LBCL [19,22].

We have analysed our case of a patient with NSHL, presenting a distinct focal area of proliferation composed of large monomorphous NHL cells in the anterior mediastinum. On immunohistochemical analysis, typical Reed-Sternberg cells expressed CD 30, CD 15 and MUM1 antigens, but lacked CD20 and Pax-5 immunoreactivity. The large monomorphous NHL cells reacted with the antibodies CD20, CD79a, MUM1 and Pax-5, showing a B-cell-related phenotype, and no other expression of the antigens was expressed by Reed-Sternberg cells expect MUM1 protein. Unfortunately, we could not perform further molecular investigations in order to determine whether a clonal relationship between the Reed-Sternberg cells and the DLBCL cells does exist in our patient. Thus, we can only offer speculations on this intriguing issue according to the existing literatures. Two main possibilities may be considered, as follows in Table 3.

**Table 3:** Clinical features of previously published cases of classical Hodgkin lymphoma with DLBCL

**NO**.	Clonal identity	Component	CD3	CD20	CD15	CD30	Immunostaining Results		ISH Results		EBER
							Igκ	Igλ		Igλ	
1	NO	RS cells	-	-	+	+	-	-	NA	NA	NA
		LBCL cells	-	+	-	-	-	-	NA	NA	NA
2	Yes	RS cells	-	-	+	+	NA	NA	NA	NA	+
		LBCL cells	-	+	-	-	NA	NA	NA	NA	-
3	Yes	RS cells	-	-	+	+	-	-	NA	NA	-
		LBCL cells	-	+	-	+	-	-	NA	NA	-
4	Yes	RS cells	-	-	+/-	+	NA	NA	NA	NA	-
		LBCL cells	-	+	-	-	NA	NA	NA	NA	-
5	NI	RS cells	-	+	+	+	+	+	-	-	NA
		LBCL cells	-	+	-	-	-	-	+	-	NA
6	NI	RS cells	-	-	+	+	+	+	-	-	NA
		LBCL cells	-	+	-	-	+	+	+	-	NA
7	Yes?	RS cells	-	-	+	+	-	-	NA	NA	-
		LBCL cells	-	+	-	-	-	+	NA	NA	-

Abbreviations: ISH, in situ hybridization; NA, not available; NI, not indicated

First, both NSHL and DLBCL originate from a common precursor B cell, that is, the same abnormal B cell gives rise to the different neoplastic disorders through distinct bilogical pathways. Bellan et al reported that immunoglobulin heavy chain gene (IgH) rearrangement was found in the V_H _region in Reed-Sternberg cells and LBCL cells. A repeated V_H4_D_H3_J_H4 _segment was observed in Reed-Sternberg cells, while a repeated V_H3_D_H3_J_H4 _segment was found in LBCL cells. The two populations showing the same J_H _and D_H _segments with no variation from the respective sequence indicated they derived from a common B cell origin [14]. In addition, clonally related V_κ_gene rearrangement was also found in both Reed-Sternberg cells and DLBCL cells which revealed a common lymphoma precursor [15]. During the course of neoplastic progression, both NSHL and DLBCL were assumed to share one or more transforming events, which might play an important role in the up-regulation or down-regulation of tumor associated antigens. For example, Pax-5 is an important B-cell transcription factor and expressed regularly in the Reed-Sternberg cells of HL. But this antigen could be lost in the Reed-Sternberg cells, which was caused by some transforming event in the tumorigeness of CL [13]. Epstein-Barr virus (EBV) was identified in a subset of cases of HL and in some NHL, particularly those associated with immunodeficiency [23]. However, EBV infection did not seem to be the primary event in this tumorigeness by assessing the data of table 3. Second, Paulli et al suggested the NSHL and DLBCL components were totally unrelated and their occurrence together within the same lymph node simply represented a coincidental occurrence [12]. However, this result might be inconclusive which was supported by immunohistochemical research and lacked the molecular studies.

Primary mediastinal large B-cell lymphoma (PMBCL) is the most critical disease in distinguishing with DLBCL of CL. PMBCL is currently classified as a subtype of diffuse large B-cell lymphoma, which has distinct clinical and molecular features, many of which are similar with that of NSHL [24]. PMBCL shows the clinical presentation of a large anterior mediastinal mass in a young female, but it does not express immunoglobulin [24-26]. In our present study, large B-cell lymphoma cells expressed Igλ which could help us differ it from PMBCL. In addition, there is a lack or defective expression of HLA class I or II molecules and an over-expression of MAL or interleukin-4 inducible gene FIG1 is always found in PMBCL [27]. Secondly, mediastinal gray zone lymphoma (MGZL), which represents two kinds of manifestation, should also be considered in differential diagnosis. In some cases, the histologic appearance is more suggestive of MLBCL, but the immunohistochemical features are characteristic of classical HL with expression of CD15 and CD30, and weak or absent CD20 positivity. In other cases, the histology is more reminiscent of classical HL, but the immunophenotype is more suggestive of a DLBCL. In our case, morphologic and immunohistochemical features have shown two different neoplasms. The Reed-Sternberg cells were positive for CD30, CD15 but negative for CD20, CD79a, CD45, while large B-cell lymphoid cells showed expression of CD20, CD79a, CD45 and absence of CD30, CD15. Other histological differential diagnoses, including leukocythemia, carcinoma metastaticum, sarcoma, reactive lymphoid proliferation and collision tumor [28] should be cautiously considered. Careful observation under the microscope and properly performed immunohistochemistry could be helpful for a correct diagnosis [2].

There is no evidence that the clinical course of CL differs significantly from that of discordant lyomphoma or lymphoma in general when the component with the poorest prognosis is taken into consideration. Although the case is rare and the number of reported cases is small, most scholars suggest that the prognosis is depended by the unfavorable component of CL. Therefore, therapeutic decision should be based on this ingredient [2,29]. The treatment for the patient we presented was CHOP chemotherapy and adjuvant radiation therapy after surgery. The necessary follow-up examination among thirty three months disclosed there was no palindromic lesion.

## Conclusion

We report a rare case of CL, combination of nodular sclerosing Hodgkin lymphoma and diffuse large B-cell lymphoma, arising in the anterior mediastinum. By molecular techniques, many scholars have proved the two different ingredients tend to origin from the same clonal progenitor cell. EBV infection is not essential transforming event during the course of tumorigenesis. The contribution of immunohistochemstry plays an important role in differential diagnoses. Therapeutic decision of CL should be based on the component of which malignant degree is higher.

## Consent

Written informed consent was obtained from the patient for publication of this case report and any accompanying images. A copy of the written consent is available for review by the Editor-in-Chief of this journal.

## Competing interests

The authors declare that they have no competing interests.

## Authors' contributions

YG and KL designed the study, performed the histological evaluation, and drafted the manuscript.

QG participated histological diagnosis and revising the manuscript.

ZQ was involved in literature search and preparing the material.

WW and JL participated in immunohistochemical evaluation.

All authors read and approved the final manuscript.

## References

[B1] National Cancer Institute sponsored study of classifications of non-Hodgkin's lymphomas summary and description of a working formulation for clinical usage. The Non-Hodgkin's Lymphoma Pathologic Classification Project Cancer 1982 49 2112 2135 6896167 10.1002/1097-0142(19820515)49:10<2112::aid-cncr2820491024>3.0.co;2-2

[B2] Kim H Hendrickson R Dorfman RF Composite lymphoma Cancer 1977 40 959 976 10.1002/1097-0142(197709)40:3<959::AID-CNCR2820400302>3.0.CO;2-3 332325

[B3] Dargent JL Lespagnard L Meiers I Bradstreet C Heimann P De Wolf-Peeters C Composite follicular lymphoma and nodular lymphocyte predominance Hodgkin's disease Virchows Arch 2005 447 778 780 10.1007/s00428-005-0008-1 15983819

[B4] Caleo A Sánchez-Aguilera A Rodríguez S Dotor AM Beltrán L de Larrinoa AF Menárguez FJ Piris MA García JF Composite Hodgkin lymphoma and mantle cell lymphoma: two clonally unrelated tumors Am J Surg Pathol 2003 27 1577 1580 10.1097/00000478-200312000-00012 14657719

[B5] Kaleem Z McGuire MH Caracioni AC Leonard RL Pathan MH Lessmann EA Chan WC Composite B-cell and T-cell non-Hodgkin lymphoma of the tibia Am J Clin Pathol 2005 123 215 221 10.1309/P6LUADHRL9TXFW2M 15842045

[B6] Kim H Composite lymphoma and related disorders Hematopathology 1993 99 445 451 10.1093/ajcp/99.4.445 8475911

[B7] Custer RP Pitfalls in the diagnosis of lymphoma and leukemia from pathologist's point of view Proceedings of Second National Conference--New York American Cancer Society 1954 554 557

[B8] Thirumala S Esposito M Fuchs A An unusual variant of composite lymphoma: a short case report and review of the literature Arch Pathol Lab Med 2000 124 1376 1378 10975943 10.5858/2000-124-1376-AUVOCL

[B9] Hoppe RT Histologic variation in non-Hodgkin's lymphomas: commentary Cancer Treat Rep 1981 65 11 12 7296559

[B10] Kim H Dorfman RF Morphological studies of 84 untreated patients subjected to laparotomy for the staging of non-Hodgkin's lymphoma Cancer 1974 33 657 674 10.1002/1097-0142(197403)33:3<657::AID-CNCR2820330311>3.0.CO;2-D 4592903

[B11] Hell K Hansmann ML Pringle JH Lauder I Fischer R Combination of Hodgkin's disease and diffuse large cell lymphoma: an in situ hybridization study for immunoglobulin light chain messenger RNA Histopathology 1995 27 491 499 10.1111/j.1365-2559.1995.tb00319.x 8838328

[B12] Paulli M Rosso R Kindl S Boveri E Sirchi M De Medici A Invernizzi R Magrini U Nodular sclerosing Hodgkin's disease and large cell lymphoma. Immunophenotypic characterization of a composite case Virchows Arch A Pathol Anat Histopathol 1992 421 271 275 10.1007/BF01611185 1384224

[B13] Huang Q Wilczynski SP Chang KL Weiss LM Composite recurrent Hodgkin lymphoma and diffuse large B-cell lymphoma. One clone, Two faces Am J Clin Pathol 2006 126 222 229 10.1309/DDGLWRV3KR9164G7 16891197

[B14] Bellan C Lazzi S Zazzi M Lalinga AV Palummo N Galieni P Marafioti T Tonini T Cinti C Leoncini L Pileri SA Tosi P Immunoglobulin Gene Rearrangement Analysis in Composite Hodgkin Disease and Large B-Cell Lymphoma: Evidence for Receptor Revision of Immunoglobulin Heavy Chain Variable Region Genes in Hodgkin-Reed-Sternberg Cells? Diagnostic Molecular Pathology 2002 11 2 8 10.1097/00019606-200203000-00002 11854595

[B15] Rosenquist R Menestrina F Lestani M Küppers R Hansmann ML Bräuninger A Indications for peripheral light-chain revision and somatic hypermutation without a functional B-cell receptor in precursors of a composite diffuse large B-cell and Hodgkin's lymphoma Lab Invest 2004 84 253 262 10.1038/labinvest.3700025 14688797

[B16] Bräuninger A Hansmann ML Strickler JG Dummer R Burg G Rajewsky K Küppers R Indentification of common germinal-center B-cell precursors in two patient with both Hodgkin's disease and non-Hodgkin's lymphoma N Eng J Med 1999 340 1239 1246 10.1056/NEJM199904223401604 10210707

[B17] Savage KJ Monti S Kutok JL Cattoretti G Neuberg D De Leval L Kurtin P Dal Cin P Ladd C Feuerhake F Aguiar RC Li S Salles G Berger F Jing W Pinkus GS Habermann T Dalla-Favera R Harris NL Aster JC Golub TR Shipp MA The molecular signature of mediastinal large B-cell lymphoma differs from that of other diffuse large B-cell lymphomas and shares features with classical Hodgkin lymphoma Blood 2003 102 3871 3879 10.1182/blood-2003-06-1841 12933571

[B18] Fend F Quintanilla-Martinez L Kumar S Beaty MW Blum L Sorbara L Jaffe ES Raffeld M Composite Low Grade B-Cell Lymphomas with Two Immunophenotypically Distinct Cell Populations Are True Biclonal Lymphomas A Molecular Analysis Using Laser Capture Microdissection American Journal of Pathology 1999 154 1857 1866 10.1016/S0002-9440(10)65443-0 10362812 PMC1866627

[B19] Greiner TC Gascoyne RD Anderson ME Kingma DW Adomat SA Said J Jaffe ES Nodular lymphocyte-predominant Hodgkin's disease associated with large- cell lymphoma: analysis of Ig gene rearrangements by V-J polymerase chain reaction Blood 1996 88 657 666 8695813

[B20] Schmitz R Renné C Rosenquist R Tinguely M Distler V Menestrina F Lestani M Stankovic T Austen B Bräuninger A Hansmann ML Küppers R Insights into the multistep transformation process of lymphomas: IgH-associated translocations and tumor suppressor gene mutations in clonally related composite Hodgkin's and non-Hodgkin's lymphomas Leukemia 2005 19 1452 1458 10.1038/sj.leu.2403841 15973455

[B21] Küppers R Sousa AB Baur AS Strickler JG Rajewsky K Hansmann ML Common germinal-center B-cell origin of the malignant cells in two composite lymphomas, involving classical Hodgkin's disease and either follicular lymphoma or B-CLL Mol Med 2001 7 285 292 11474574 PMC1950043

[B22] Ohno T Huang JZ Wu G Park KH Weisenburger DD Chan WC The tumor cells in nodular lymphocyte-predominant Hodgkin disease are clonally related to the large cell lymphoma occurring in the same individual. Direct demonstration by single cell analysis Am J Clin Pathol 2001 116 506 511 10.1309/KY8C-LCYN-QHJ6-4C6R 11601135

[B23] Kingma DW Medeiros LJ Barletta J Raffeld M Mann RB Ambinder RF Jaffe ES Epstein-Barr virus is infrequently identified in non-Hodgkin's lymphomas associated with Hodgkin's disease Am J Surg Pathol 1994 18 48 61 10.1097/00000478-199401000-00005 8279628

[B24] Jaffe ES Harris NL Stein H World Health Organization classification of tumors Pathology and genetics of tumors of hematopoietic and lymphoid tissues 2001 IARC Press: Lyon, France

[B25] Lamarre L Jacobson JO Aisenberg AC Harris NL Primary large cell lymphoma of the mediastinum. A histologic and immunophenotypic study of 29 cases Am J Surg Pathol 1989 13 730 739 10.1097/00000478-198909000-00002 2788371

[B26] Rosenwald A Wright G Leroy K Yu X Gaulard P Gascoyne RD Chan WC Zhao T Haioun C Greiner TC Weisenburger DD Lynch JC Vose J Armitage JO Smeland EB Kvaloy S Holte H Delabie J Campo E Montserrat E Lopez-Guillermo A Ott G Muller-Hermelink HK Connors JM Braziel R Grogan TM Fisher RI Miller TP LeBlanc M Chiorazzi M Zhao H Yang L Powell J Wilson WH Jaffe ES Simon R Klausner RD Staudt LM Molecular diagnosis of primary mediastinal B cell lymphoma identifies a clinically favorable subgroup of diffuse large B cell lymphoma related to Hodgkin lymphoma J Exp Med 2003 198 851 862 10.1084/jem.20031074 12975453 PMC2194208

[B27] Faris JE LaCasce AS Primary mediastinal large B-cell lymphoma Clin Adv Hematol Oncol 2009 7 125 133 19367254

[B28] El Demellawy D Ross C Sur M Alowami S Synchronously diagnosed lymph nodal collision tumor of malignant melanoma and chronic lymphocytic leukemia/small lymphocytic lymphoma: case report Diagn Pathol 2007 2 34 10.1186/1746-1596-2-34 17760975 PMC2040134

[B29] Travis LB Gonzalez CL Hankey BF Jaffe ES Hodgkin's disease following non-Hodgkin's lymphoma Cancer 1992 69 2337 2342 10.1002/1097-0142(19920501)69:9<2337::AID-CNCR2820690923>3.0.CO;2-K 1562981

